# A valuable subarachnoid space named the occipito-atlantal cistern

**DOI:** 10.1038/s41598-023-38825-z

**Published:** 2023-07-26

**Authors:** Yun-Fei Li, Rui-Xue Wei, Kai-Qi Yang, Gary D. Hack, Yan-Yan Chi, Wei Tang, Xue-Jun Sui, Meng-Liang Zhang, Hong-Jin Sui, Sheng-Bo Yu

**Affiliations:** 1 Department of Anatomy, College of Zhongshan, Dalian Medical University, Dalian, 116085 China; 2 Second Affiliated Hospital, Dalian Medical University, Dalian, 116052 China; 3 Department of Anatomy, College of Basic Medical Sciences, Dalian Medical University, Dalian, 116044 China; 4 Department of Advanced Oral Sciences and Therapeutics, University of Maryland School of Dentistry, Baltimore, MD 21201 USA; 5 Dalian Hoffen Preservation Technique Institution, Dalian, 116052 China; 6 Department of Molecular Medicine, University of Southern Denmark, 5000 Odense C, Denmark; 7 China Denmark Joint Research Center for Biological Plastination Technique, Dalian Medical University, Dalian, 116044 China

**Keywords:** Biological techniques, Anatomy

## Abstract

The cisterna magna has been defined as the space between the inferior margin of the cerebellar vermis to the level of the foramen magnum, while an enlarged dorsal subarachnoid space at the occipito-cervical junction extending from the foramen magnum to the upper border of the axis (C2) is still ignored. Recently, the myodural bridge complex is proved to drive the cerebral spinal fluid flowing via this region, we therefore introduce the “occipito-atlantal cistern (OAC)” to better describe the subarachnoid space and provide a detailed rationale. The present study utilized several methods, including MRI, gross anatomical dissection, P45 sheet plastination, and three-dimensional visualization. OAC was observed to be an enlarge subarachnoid space, extending from the foramen magnum to the level of the C2. In the median sagittal plane, OAC was a funnel shape and its anteroposterior dimensions were 15.92 ± 4.20 mm at the level of the C0, 4.49 ± 1.25 mm at the level of the posterior arch of the C1, and 2.88 ± 0.77 mm at the level of the arch of the C2, respectively. In the median sagittal plane, the spino-dural angle of the OAC was calculated to be 35.10 ± 6.91°, and the area of OAC was calculated to be 232.28 ± 71.02 mm^2^. The present study provides OAC is a subarachnoid space independent from the cisterna magna. Because of its distinctive anatomy, as well as theoretical and clinical significance, OAC deserves its own name.

## Introduction

The cerebral spinal fluid (CSF) flows out of the fourth ventricle via the median and lateral apertures, and then drains into the posterior cerebellomedullary cistern (PCC). The PCC, also called the cisterna magna, is located between the dorsal surface of the medulla oblongata and the inferior aspect of the cerebellum. The upper horn of the PCC communicates with the fourth ventricle via the median and lateral apertures. The base of the PCC has previously been described as being at the level of the foramen magnum (FM, C0)^1^. However, the posterior cervical subarachnoid space from the level of the arch of the atlas (C1) to the foramen magnum has been considered as an aspect of the cisterna magna by some^2^. It’s unclear whether there is a subarachnoid space located at the occipito-cervical junction (OCJ) distinct from the cisterna magna.

The OCJ is one of the most complicated and flexible portions of the human body. The circulation of cerebrospinal fluid within the PCC has been reported to be affected by motions of the head and neck. It also has been proposed that the myodural bridges (MDB) previously described in the suboccipital region can place tension on the upper part of the cervical spinal dura mater “pumping” the CSF flowing during head and neck movements^3–10^. Recently, Xu et al.^11^ found that the head rotation significantly changed CSF flow rate at the level of atlas. CSF located in the ventricles initially drains into the PCC and then spreads to the subarachnoid space over the brain and the spinal cord via a kinetic mechanism^12^. Therefore, future researches involving CSF circulation should consider the upper part of the cervical subarachnoid space.

In clinic, Arnold-Chiari type 1 malformation often results in abnormal CSF pressure and flow rate, which leads to changes in the circulation patterns of the CSF near the OCJ. And syringomyelia occurs when cerebrospinal fluid infiltrates or impacts the spinal cord^13–17^. These changes to CSF circulation near the OCJ may play an important role in the pathogenesis of Arnold-Chiari type 1 malformation. Currently, the PCC above the FM, and the subarachnoid space of the upper cervical spine below the FM, are considered as separate entities when considering treatments for pathologies of the head and neck^18^.

In the present study, the subarachnoid space in the upper cervical spine was observed and measured. We propose that the best name for this space is the “occipito-atlantal cistern”, which will hopefully assist anatomical, physiological, pathological research, and clinical studies in the region of the OCJ.

## Materials and methods

This study was approved by the Ethics Committee for Research at the Basic Medical College of Dalian Medical University. All methods were performed in accordance with relevant guidelines and regulations.

### Experimental subjects

Sixty cases of head and neck MR images were analyzed (23 males, 41.74 ± 12.44 years, 37 females, 39.24 ± 14.40 years), which were derived from patients without obvious head and neck lesions. Six adult cadaveric head and neck specimens fixed with 10% formalin in the present study were obtained from the Body and Organs Donation Center of Dalian Medical University. Before the donors involved in the current study passed away, written informed consent was obtained from them in compliance with the ethical committee's rules. Three specimens were utilized for gross anatomical dissection, and the other three were used for P45 sheet plastination studies, which were prepared at the Dalian Hoffen Preservation Technique Institution. In addition, one set of head and neck digital data was reconstructed, which was obtained from the Visible Korean project, an anatomical photographic digital database^19^.

### Gross anatomical dissections

During dissection in the suboccipital region, the soft tissue, the posterior wall of the vertebral canal, and the basilar and lateral parts of the occipital bone were separated layer by layer to open the cranial cavity and the vertebral canal, to expose the cranial and spinal dura mater. And then the dura mater was incised longitudinally along the posterior median line to explore the arachnoid mater in the posterior cranial fossa to the third cervical vertebra. After that, a cruciform incision was made on the arachnoid mater to open and observe shape and contents of the subarachnoid space in the region of the OCJ.

### P45 sheet plastination

P45 sheet plastination of three head and neck specimens was carried out, to obtain sagittal, coronal, and horizontal sections. The procedures involved in the P45 sheet plastination, including slicing, bleaching, dehydration, forced impregnation, and curing have been described in current literature^8,20,21^. The slice thickness in the present study was 3 mm and the subarachnoid space adjacent to the OCJ was observed in situ.

### MRI imaging

**Figure 1:**
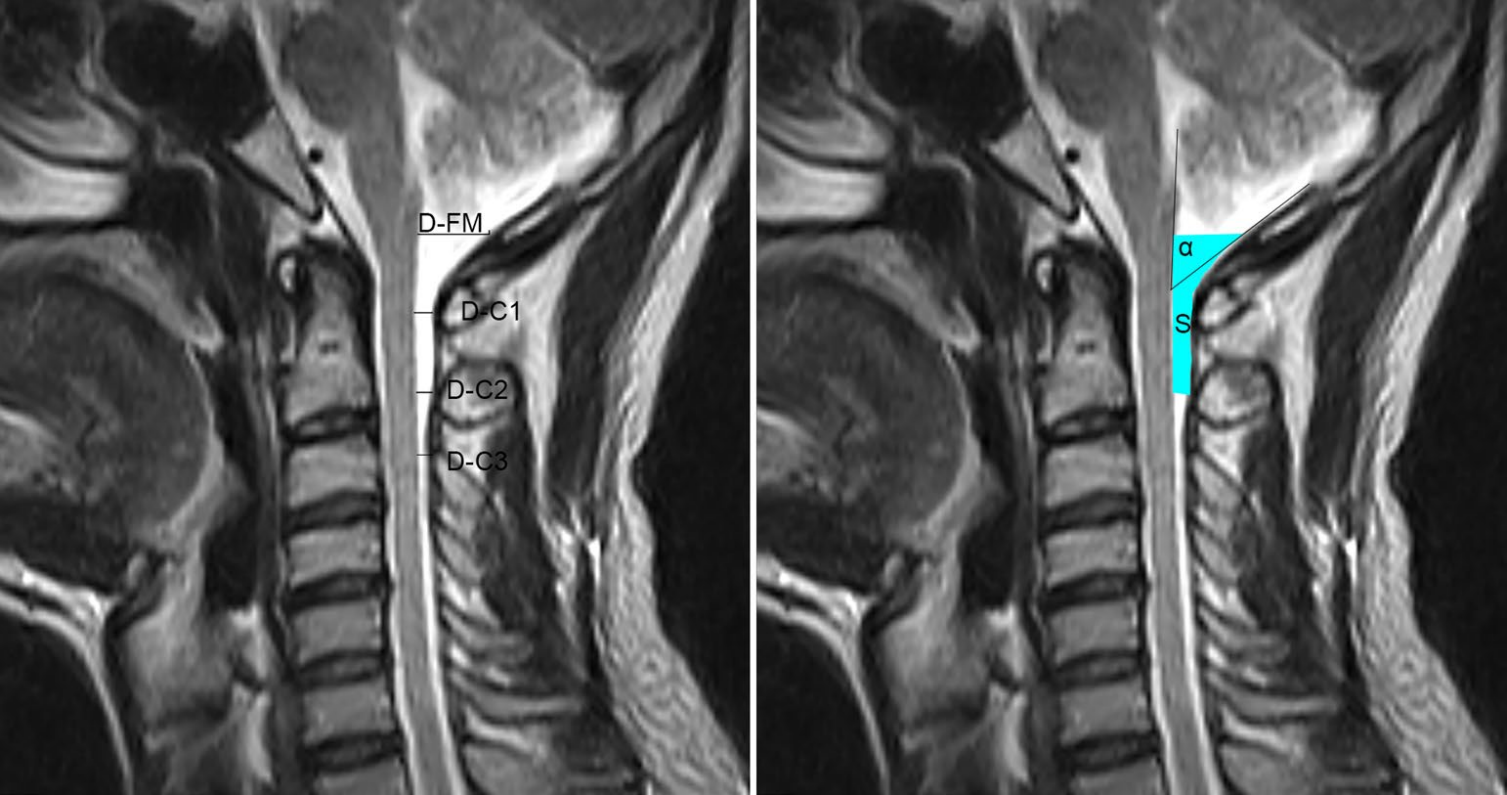
Median sagittal MRI of the head and neck showing the measurement parameters used to describe the enlarged subarachnoid space. D-FM, the anteroposterior extension of the subarachnoid space at the level of the foramen magnum (C0), measured horizontally from the posterior rim of foramen magnum (C0) to the dorsum of the medulla oblongata; D-C1, the anteroposterior extension of the subarachnoid space at the level of the atlas (C1), measured horizontally from the anterior point of the inner-convex surface of the arch of the atlas (C1) to the dorsum of the spinal cord; D-C2, the anteroposterior extension of the subarachnoid space at the level of the axis (C2), measured horizontally from the anterior point of the inner-convex surface of the arch of the axis (C2) to the dorsum of the spinal cord; D-C3, the anteroposterior extension of the subarachnoid space at the level of the third cervical vertebra (C3), measured horizontally from the anterior point of the inner-convex surface of the arch of the third cervical vertebra (C3) to the dorsum of the spinal cord; The spino-dural angle (SDA, α) of the enlarged subarachnoid space, which is formed by the dorsal side of the spinal cord and the dura extending a line passing through the posterior rim of the foramen magnum (C0) and the inner surface of the posterior arch of the atlas (C2); The median sagittal area (MSA, S) of the enlarged subarachnoid space dorsal to the spinal cord, calculated from the level of the foramen magnum (C0) to the level of the second cervical vertebra (C2) on the sagittal plane.

All MRI study underwent in the supine position. The subarachnoid space posterior to the medulla oblongata and the spinal cord were observed in the median sagittal head and neck MR images (Tesla parameter: 0.35 T). Adobe Bridge CC 2018 software was used to measure the horizontal distances of the dorsal subarachnoid space at the level of the FM (D-FM), the posterior arch of the atlas (D-C1), the arch of the axis (D-C2), and the arch of the third cervical vertebra (D-C3) respectively (see left side of Fig. 1). Two other parameters were also calculated. The spino-dural angle (SDA) was defined by the dorsal side of the spinal cord and a straight line passing through the posterior brim of the FM and the inner surface of the posterior arch of the atlas. The median sagittal area (MSA) of the dorsal subarachnoid space, from the level of the FM to the level of the arch of the axis in the median sagittal plane was also calculated (see right side of Fig. 1).

### Three-dimensional visualization

Transverse sections, from the level of the posterior cranial fossa to the level of the third cervical vertebra, were obtained from the photographic digital dataset. The structures of interest were segmented in the selected images, which included the tonsils of the cerebellum, the medulla oblongata, the dura mater, the spinal cord, the PCC, the subarachnoid space, the vertebral artery, the spinal nerve roots, and the hypoglossal nerve root. Mimics Innovation Suite 18.0 software was employed for the 3D-reconstructions. The set pixel size and layer thickness were maintained at x = 0.1 mm, y = 0.1 mm, z = 0.2 mm, for the 3D model calculations. The enlarged subarachnoid space (ESS) observed posterior to the lower part of the medulla oblongata and upper part of the cervical portion of the spinal cord was visualized in the 3D images.

## Results

### Gross anatomical dissection

**Figure 2:**
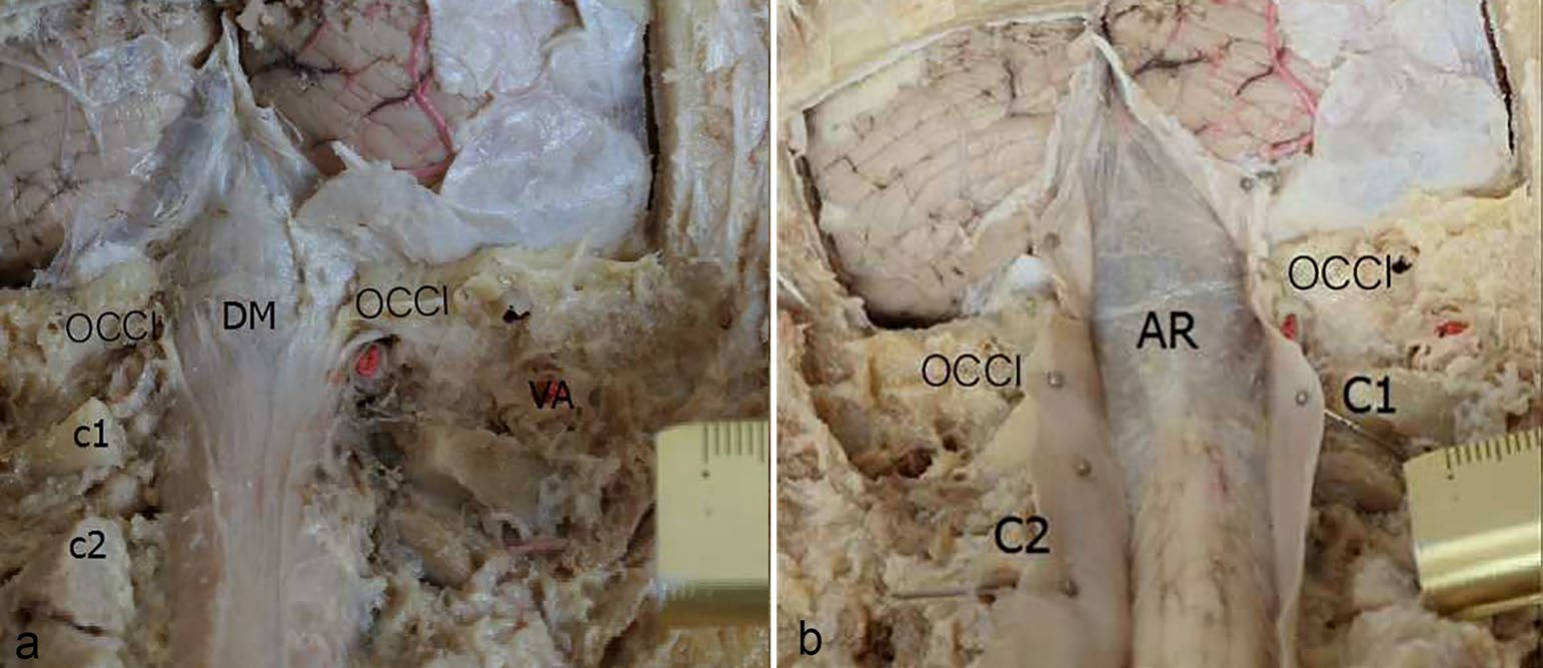
A posterior view of the posterior cranial fossa and the upper cervical vertebral canal (**a**, showing the shape and extension of the dura mater; **b**, showing the shape and extension of the arachnoid mater). (**a**) The cranial dura mater was observed to be continuous with the spinal dura mater when passing over the foramen magnum. (**b**) the arachnoid mater covered the subarachnoid space above and below the foramen magnum and appeared as a translucent structure in the diamond area with its upper half gradually narrowing towards the vermis of the cerebellum and its lower half gradually extending inferiorly to the level of the atlas (C2). The enlarged subarachnoid space existed below the foramen magnum extending inferiorly to the level of the axis (C2), whose upper aspect was connected to the cisterna magna. OCCI, occipital bone and the foramen magnum; C1, atlas; C2, axis; DM: dura mater; AR, arachnoid mater.

**Figure 3:**
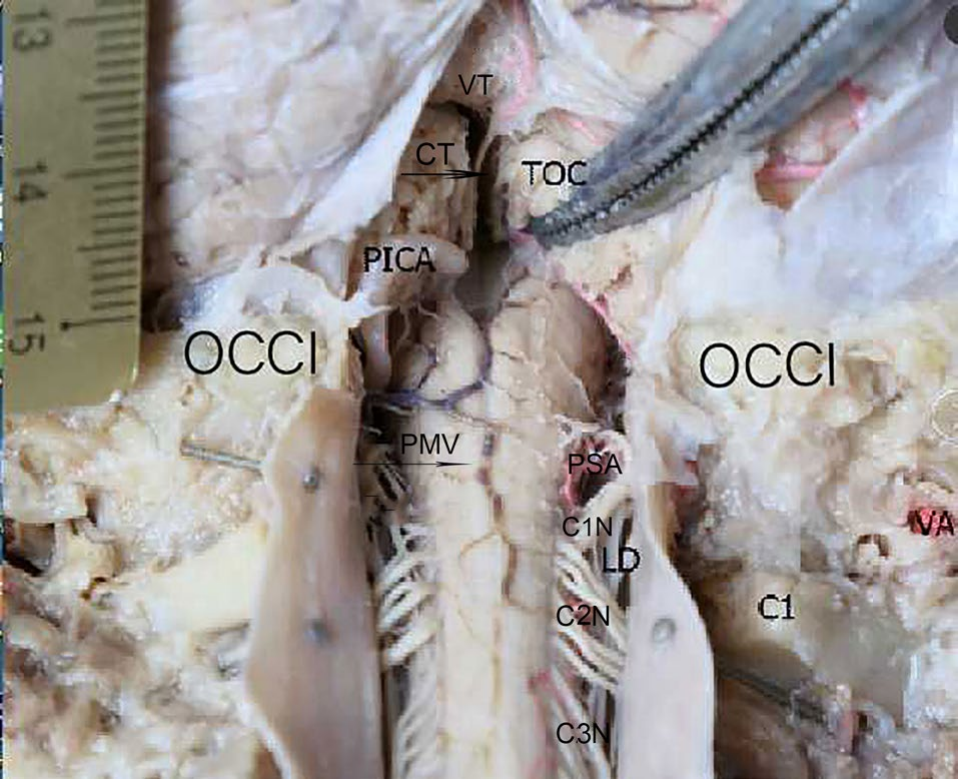
Posterior view of the cerebellum, medulla oblongata, and the upper cervical spine with the arachnoid mater having been removed. The enlarged subarachnoid space was exposed, and its was observed to be below the foramen magnum (C0) and dorsal to a series of structures consisting of the C1-3 spinal segments, their spinal nerve roots, and the denticulate ligaments. VT, the tuber of the vermis; OCCI, occipital bone and the foramen magnum; C1, the atlas; LD, the denticulate ligament of the spinal cord; PICA, the posterior inferior cerebellar artery; VA, the vertebral artery; TOC, the tonsil of the cerebellum; CT, the choroid tissue of fourth ventricle; PSA, the posterior spinal artery; PMV, the posterior median vein of the spinal cord; C1N, the root of the first cervical spinal nerve; C2N, the root of the second cervical spinal nerve; C3N, the root of the third cervical spinal nerve.

Dissection of the three of head and neck specimens clearly evidenced the normal structures contained in the posterior cranial fossa and the upper cervical spine (Figs. 2, 3). After the cranial and spinal dura mater were exposed and removed (Fig. 2a), above and below the level of the FM, the arachnoid mater presented as a translucent, diamond-shaped area, which gradually narrowed and closed towards the vermis of cerebellum above, and gradually narrowed inferiorly to the level of the lamina of axis (Fig. 2b). After removal of the arachnoid mater, an obviously enlarged subarachnoid space was found in the OCJ. The upper half part of the space was the cisterna magna, located above the FM, and the lower half part of the space was observed below the FM and located dorsal to a series of structures, which included the C1-3 spinal segments, their spinal nerve roots, and the denticulate ligaments (Fig. 3).

### P45 sheet plastination

Structures in the occipito-cervical junction, such as the occipital bone, the cervical vertebrae, the medulla oblongata, the cerebellum, the spinal cord, the dura mater, and the subarachnoid space were clearly observed in the P45 plastinated slices.

**Figure 4:**
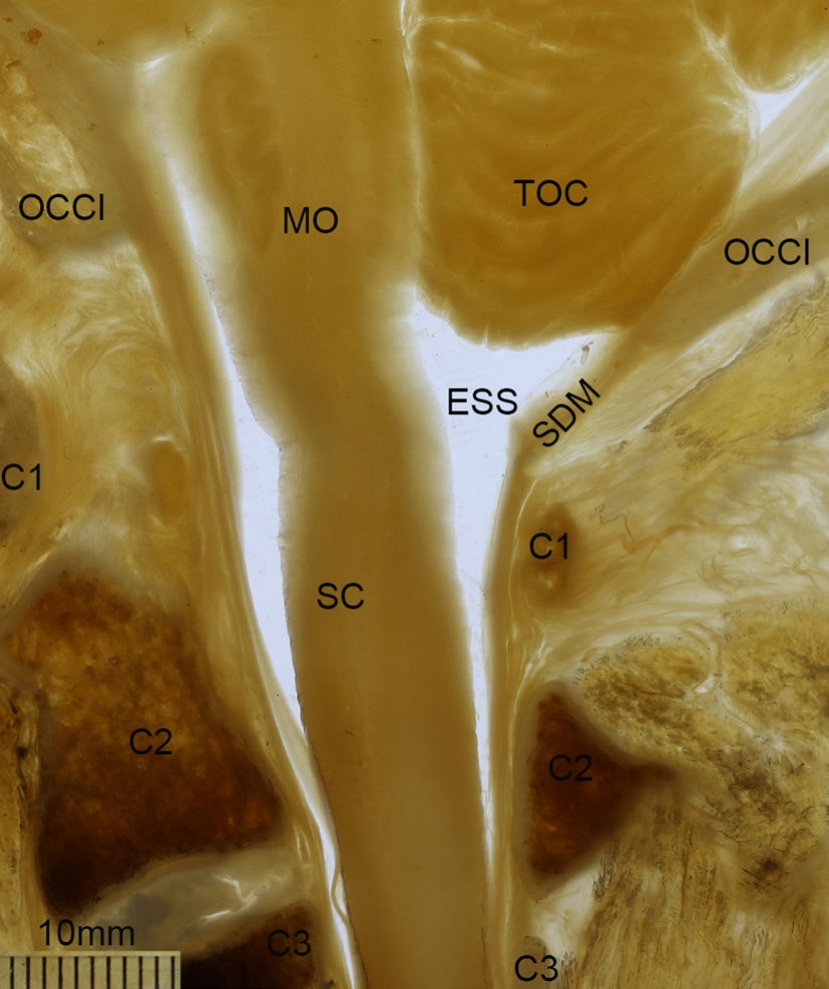
A median sagittal P45 plastinated section of the posterior cranial fossa and the upper cervical vertebral canal. The enlarged subarachnoid space was observed to be dorsal to the lower part of the medulla oblongata (MO) and the upper part of the cervical portion of the spinal cord (SC), which gradually became narrowed down to the level of axis (C2). ESS, the enlarged subarachnoid space; OCCI: occipital bone and the foramen magnum; C1, the atlas; SDM, the spinal dura mater; C3, the third cervical vertebra.

**Figure 5:**
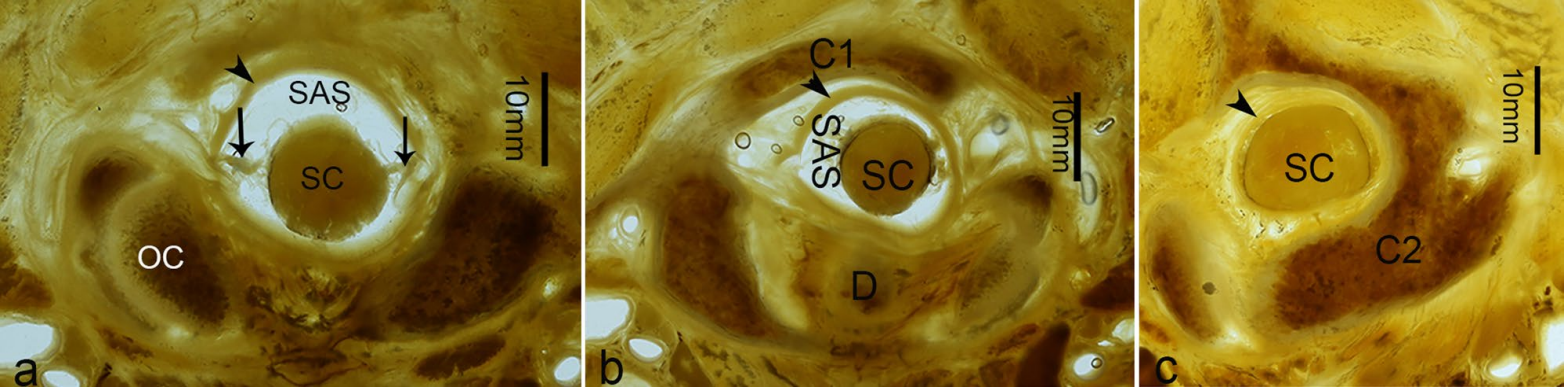
Horizontal P45 plastinated sections of the upper cervical spine. (**a**), the section through the occipital condyle; (**b**), the section at the level of the atlas (C1); (**c**), the section at the level of the axis (C2). At the level of the occipital condyle (OC), the subarachnoid space (SAS) dorsal to the spinal cord was large, the anterior border of which was constituted by the spinal cord (SC) and its nerve roots. At the level of atlas (C1), there was a relatively large subarachnoid space dorsal to the spinal cord. However, at the level of axis (C2), the subarachnoid space is not clearly seen. D, the dens of axis. Arrows, the spinal nerve roots; SDM, the spinal dura matter.

In the median sections, an enlarged subarachnoid space (ESS) was observed below the FM, dorsal to both the lower part of the medulla oblongata and the upper part of the cervical spinal cord. The roof of the ESS was at the level of the FM and the floor was narrowed down to the level of axis (C2) (Fig. 4). In the horizontal sections through the occipital condyle, just below the FM, the enlarged subarachnoid space was observed dorsal to the spinal cord (Fig. 5a), the anterior wall of which was composed of the spinal cord and its nerve roots. When at the level of the atlas (C1), there was still observed a relatively large subarachnoid space dorsal to the spinal cord (Fig. 5b), while a narrow subarachnoid space was presented, below the level of the upper border of the lamina of axis (C2) (Fig. 5c).

**Figure 6:**
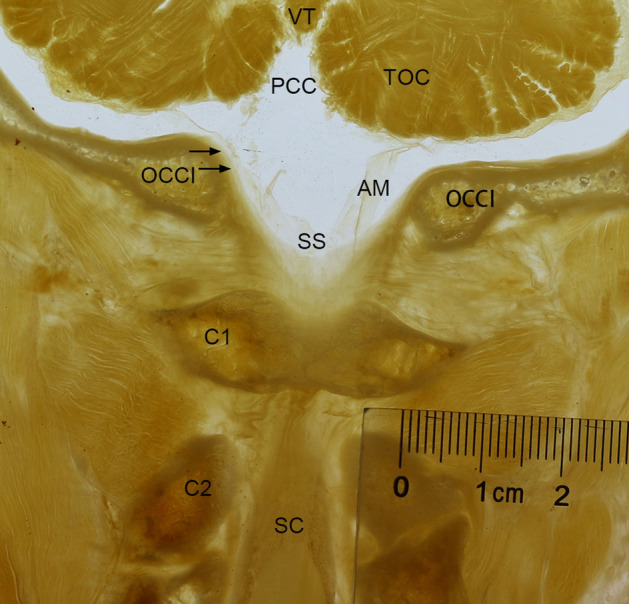
A frontal P45 plastinated section of the occipito-cervical junction through the posterior arch of the atlas (C1). There was a large and rhombic shaped cistern observed between the cerebellum and the atlas (C1). The upper aspect was the posterior cerebellomedullary cistern. The lower aspect was positioned between the foramen magnum and the atlas (C1) and was enclosed posteriorly by the spinal dura mater. OCCI, occipital bone and the foramen magnum; C2, the axis; SC, the spinal cord; AM, Arachnoid membrane; Arrows, the dura mater; SS, subarachnoid space.

In addition, in the coronal head and neck sections through the posterior arch of the atlas (C1), a large and rhombic-shaped cistern was observed between the cerebellum and the atlas. Its upper portion, namely the PCC, was located above the FM, and the lower portion of the cistern was positioned within the vertebral canal and located between the FM and the atlas (C1) (Fig. 6).

### MR imaging measurements

**Figure 7:**
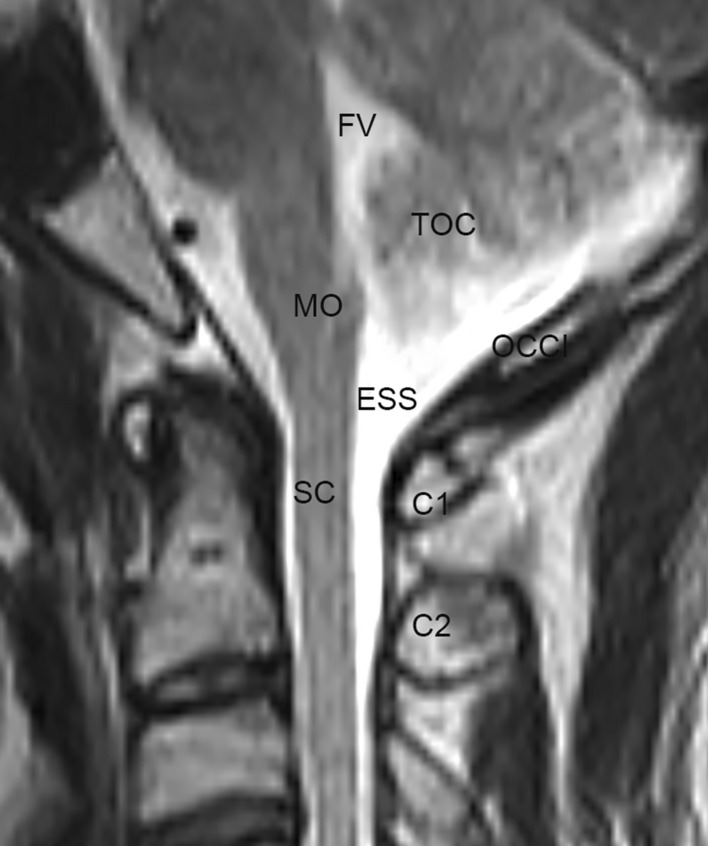
A median sagittal section of the head and neck (MRI). It was observed that the enlarged subarachnoid space was present on the dorsal side of the lower part of the medulla oblongata (MO) and the upper part of the cervical portion of the spinal cord (SC) and from the level of the foramen magnum down to the level of the axis (C2). OCCI, the occipital bone and the foramen magnum; C1, the posterior arch of the atlas; FV, fourth ventricle.

In the median sagittal head and neck sections, the ESS was located along the dorsal side of both the inferior portion of the medulla oblongata and the superior portion of the cervical spinal cord. This space extended downwards and narrowed gradually from the level of the C0 down to the level of the C2 (Fig. 7).

**Figure 8:**
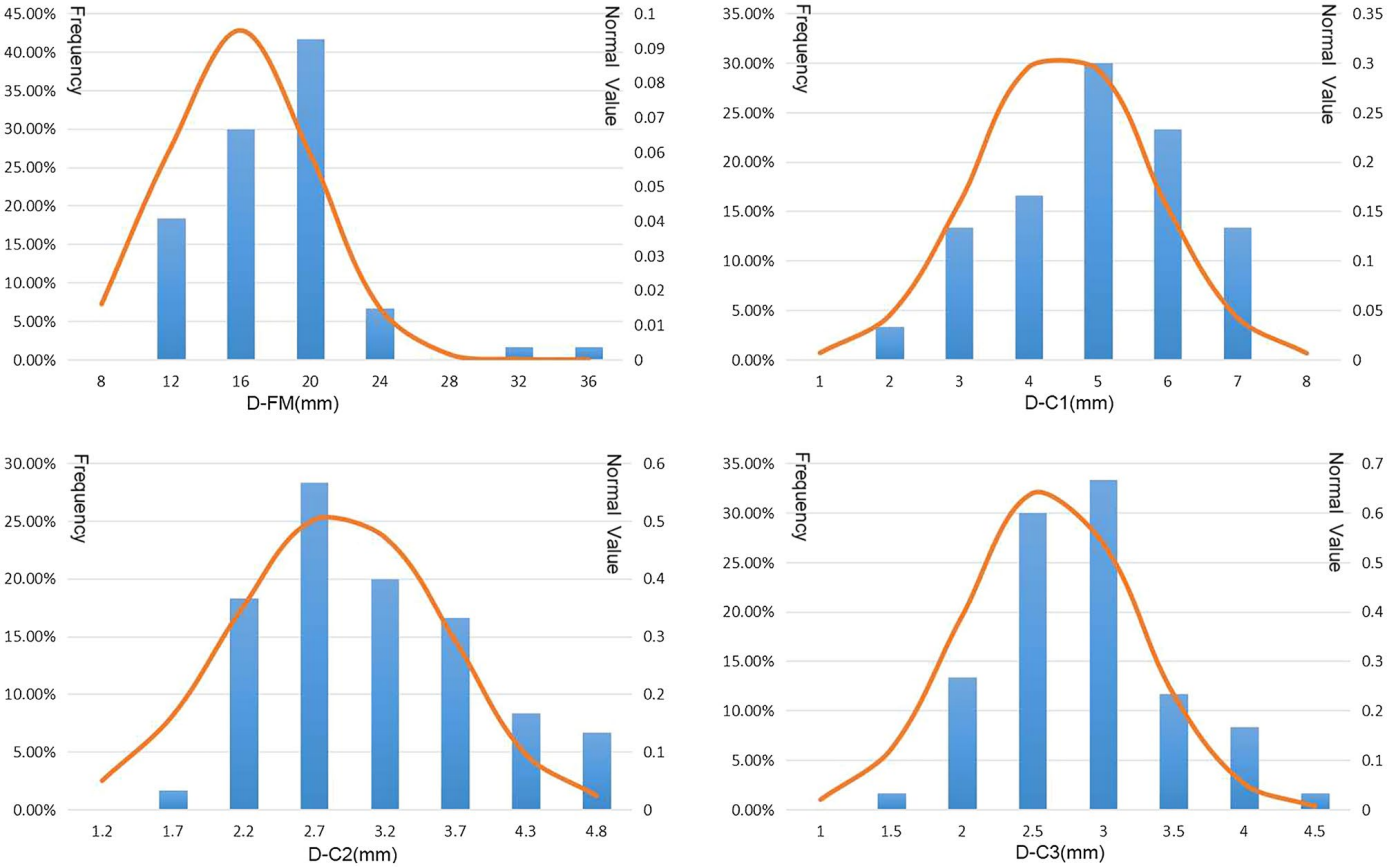
Charts displaying the frequency distribution of dimensions of the subarachnoid space, which is located dorsal to the upper part of the cervical portion of the spinal cord. (**a**), D-FM at the level of the foramen magnum (C0); (**b**), D-C1 at the level of the atlas (C1); (**c**), D-C2 at the level of the axis (C2); (**d**), D-C3 at the level of the third cervical vertebra (C3).

**Figure 9:**
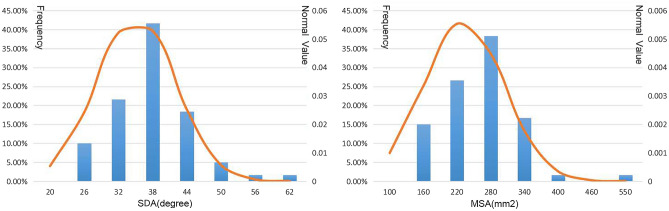
Charts of displaying the frequency distributions of the median sagittal area (MSA) and the spino-dural angle (SDA) of the subarachnoid space, which is located dorsal to the upper part of the cervical portion of the spinal cord at the occipitocervical junction (OCJ). (**a**), the SDA (α); (**b**), the MSA (S).

On the base of MRI measurements of the 60 cases, and the anteroposterior extension parameter in the median horizontal plane of the ESS was measured 15.92 ± 4.20 mm at the level of the FM (D-FM), 4.49 ± 1.25 mm at the level of the atlas (D-C1), 2.88 ± 0.77 mm at the level of the axis (D-C2), and 2.62 ± 0.61 mm at the level of the third cervical vertebra (D-C3). In the median plane, the spino-dural angle (SDA) of the ESS was 35.10 ± 6.91°, and the median sagittal area (MSA) of this space from the FM to the upper edge of the arch of the axis was 232.28 ± 71.02 mm^2^. These morphological parameters showed no significant differences between males and females, with their frequency distribution being shown in Figs. 8 and 9.

### Three-dimensional visualization

**Figure 10:**
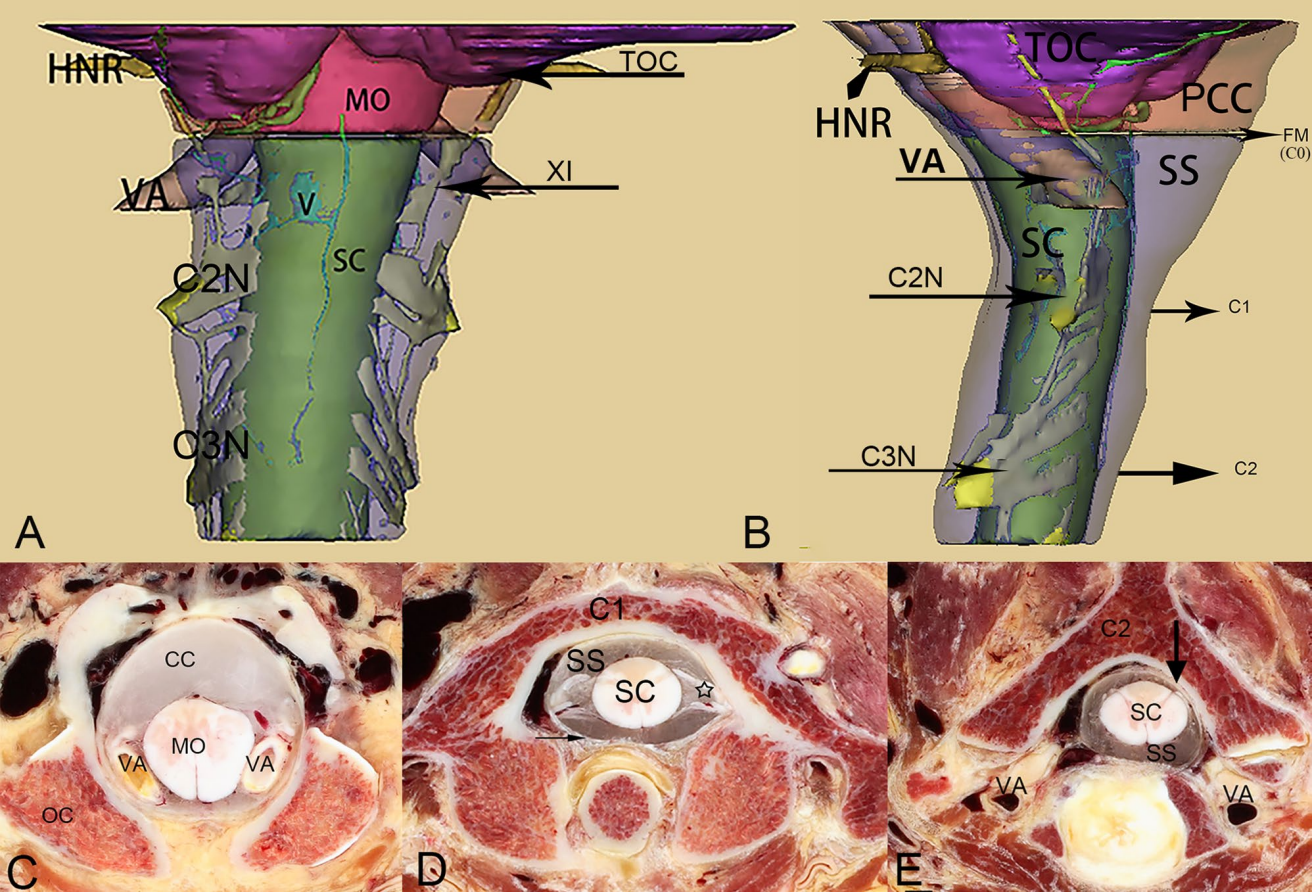
A three-dimensional model of the structures contained within both the posterior cranial fossa and the upper cervical vertebral canal (figure **A**–**E** from the same individual) [**A**, the dorsal view of the 3D model; **B**, the lateral view of the 3D model; **C**, the horizontal section through the foramen magnum (C0); **D**, the horizontal section through the atlas (C1); **E**, the horizontal section through the axis (C2)]. TOC, tonsil of the cerebellum; MO, medulla oblongata; SC, the spinal cord; PCC, the posterior cerebellomedullary cistern; SS, subarachnoid space; VA, vertebral artery; V, Vein; XI, accessory nerve; C2N, root of the second cervical spinal nerve; C3N, root of the third cervical spinal nerve; HNR, hypoglossal nerve root; FM, foramen magnum; C1, the atlas; C2, the axis; Arrowhead, dura mater.

**Figure 11:**
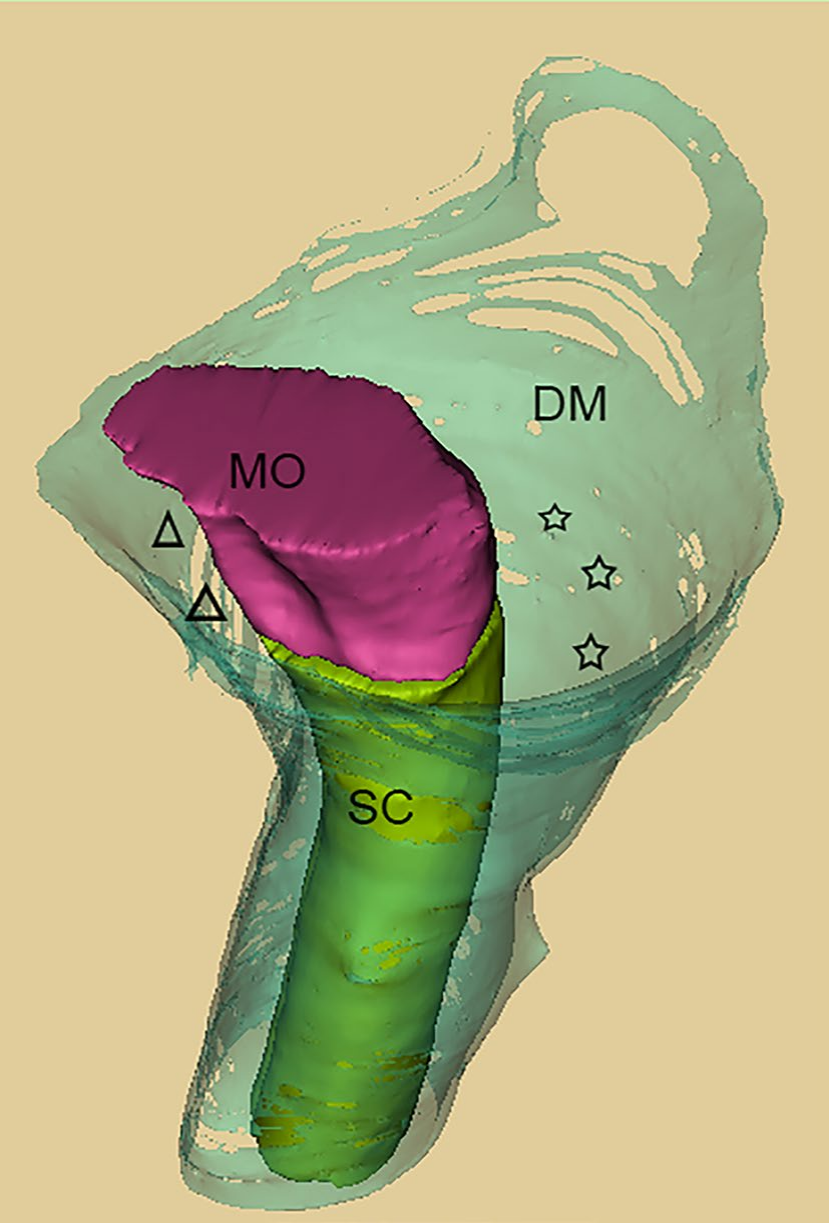
Anterior-lateral-superior view of a three-dimensional model at the occipito-cervical junction, including the medulla oblongata, the upper part of the cervical portion of the spinal cord, and the dural sac. The dural sac around the the occipito-cervical junction (OCJ) was divided into an anterior narrow part (triangles) and a posterior broad part (stars). The posterior broad part appeared as a semi funnel shaped subarachnoid space, which was dorsal to both the medulla oblongata and the upper part of the cervical portion of the spinal cord and enclosed by the dura mater both posteriorly and laterally. SC, spinal cord; MO, medulla oblongata; DM, dura mater.

The structures contained within the posterior cranial fossa and the upper cervical spine were reconstructed from the digital human dataset. The reconstructed structures included the tonsils of the cerebellum, the medulla oblongata, the spinal cord, the nerve roots, the vertebral arteries, and the dural sac (Fig. 10). The dural sac at the OCJ was subdivided into a narrow anterior part, and a broad posterior part by the medulla oblongata and spinal cord, the posterior one was an aspect of the semi-funnel shaped ESS, which was located dorsal to the medulla oblongata and the upper part of the cervical spinal cord and enclosed by the dura mater posteriorly and laterally. The superior portion of the ESS was communicated with the cisterna magna through the FM (C0). The inferior aspect of this space became narrowed gradually down to the level of the axis (C2), while it bulged posteriorly again in the posterior atlanto-axial interspace (Figs. 10, 11). On horizontal sections, the ESS was shown crescent-shaped, and at the level of FM (C0), its anterior border was formed by the medulla oblongata, the vertebral arteries, and the accessory nerves, and in the lower part of the ESS, at the level of the atlas (C1), its anterior border was composed of the spinal cord and the roots of spinal nerves. While, at the level of the axis, the subarachnoid space was observed to be minimal, as shown in Fig. 10.

## Discussion

The present study found that an enlarged subarachnoid space (ESS) located around the OCJ, whose upper aspect was communicated with the cisterna magna through the FM (C0), and whose ventral wall extended along the dorsal sides of the following structures: the C1-2 spinal segments, their spinal nerve roots, and the denticulate ligament. In the median sagittal section, the ESS appeared to be triangular in shape and was observed to be located dorsal to both the medulla oblongata and the upper portion of the cervical spinal cord. Its floor was narrowed down to the level of the axis. Furthermore, morphometric parameters of the ESS are also described in the present study.

Based on the results obtained from the dissectional observation and MRI measurements, it was believed that there is a subarachnoid space located at the OCJ distinct from the cisterna magna, and the logical name for this space is the “occipito-atlantal cistern (OAC)”. In a result, the dorsal subarachnoid space surrounding the OCJ should now be considered as consisting of two portions, the cisterna magna and the OAC. The OAC includes a significant amount of the CSF in the OCJ. This new finding has theoretical and clinical significance.

Considering CSF circulation, the OAC is an important relay center, accepting CSF from the cisterna magna, then releasing it slowly into the subarachnoid space of the vertebral canal^12^. Some scholars have reported that head rotations changed the flow rate of CSF at the OCJ^10^. In 2014, Zheng et al. proposed a functional hypothesis that the myodural bridges (MDBs) may provide power for CSF circulation^6^. In the suboccipital region, the MDBs originate from the rectus capitis posterior minor (RCPmi), the retus capitis posterior major (RCPma), and the obliquus capitis inferior (OCI) muscles, and the nuchal ligament. All of these structures were found to connect to the spinal dura mater after passing through the posterior atlanto-occipital or atlanto-axial interspaces^3,4,6–9,22–24^. Thus, during head and neck movements the posterior wall of the upper cervical portion of the dural sac would be pumped via the MDBs. Recently, researchers proposed that the myodural bridge complex could provide power for the circulation of CSF during head movements^7,10,11,13,24^. The OCJ appears to be a critical anatomical and physiological region for CSF circulation. Therefore, it becomes important to consider the CSF both above and below the FM in future studies of CSF circulation. The OAC becomes a pivotal hub for the diffusion of CSF from the cisterna magna to the subarachnoid space of the upper cervical spine over the surface of brain and spinal cord.

In clinical practice, Chiari I malformations (CIM) mainly occur at the OCJ, and syringomyelia is a common complication^13–17^. At present, its main etiology is CSF hydrodynamics and CSF pressure separation in the subarachnoid space of the OCJ^14–16^. The reduction of the subarachnoid space at the OCJ is commonly associated with CIM. The abnormal development of the posterior cranial fossa and the downward movement of the tonsils of the cerebellum result in a disruption of cerebrospinal fluid circulation and potentially hydrocephalus. With this condition, the OAC becomes narrowed which leads to an abnormal increase in the velocity of the cerebrospinal fluid flow rate which may represent the pathogenic mechanism of syringomyelia^14,16,17^. Syringomyelia may worsen and develop superiorly to lead to syringobulbia. CIM is diagnosed by MRI findings of at least 5 mm of cerebellar ectopy below the foramen magnum. The position of the tonsils and brain stem, the presence of a syrinx and other associated imaging findings suggestive of symptomatic lesions are assessed^25^. Additionally, phase-contrast magnetic resonance imaging (PC-MRI) is a useful supplement to morphological imaging for postoperative evaluation and follow-up of CIM patients^26,27^. The analysis of CSF flow dynamics focused on the ventral space between the clivus and brain stem at the level of the foramen magnum, the central aqueduct and IV ventricle, and the posterior fossa space behind the cerebellum and tonsils. Our research points out that the OAC is a crucial area connecting the subarachnoid space of brain and spinal cord. The morphological changes of the OAC and the CSF flow dynamics at its upper and lower openings should be noted in future imaging examinations. Thus, we suggest that the newly described OAC is a clinically significant subarachnoid space independent of the cisterna magna. Increased knowledge of the subarachnoid space above the upper rim of C2 provides an important anatomical basis for future clinical studies of nervous system disorders.

Previously, the cistern just below the cisterna magna was noticed by Yasargil, and it was described as “cervical subarachnoid space”^28^. There are often a median sheet of arachnoid divides the cistern into sagittal halves, and two additional paramedian septi are formed at the level of C1/2 and extend to the level of T11-12, dividing the dorsal spinal subarachnoid space longitudinally. We propose the expanded space formed by the OAC and PCC at the OCJ may act as a buffer zone for CSF dynamics stability in both cranial cavity and spinal canal, and regulate the flow of CSF through those longitudinal spaces mentioned by Yasargil. Furthermore, the OAC and the terminal cistern are enlarged subarachnoid spaces in the spine at the cephalic and caudal ends, respectively, that create a dumbbell-shaped distribution of CSF. This volume distribution may help to prevent sudden changes in CSF pressure during movement.

The study has some limitations, the foremost being the use of limited research materials. This is a small sample study, based solely on a population of northern China and South Korea. We look forward to further research expanding the number of subjects and populations. Additionally, all MRI examinations were conducted in the supine position, leading to a smaller measurement of the OAC due to the effect of gravity. And there may be slight shrinkage of the P45 plastination samples, which potentially could affect the results of the P45 section studies. Moreover, the research methods employed in this study were gross and sectional dissection. Tissue micro-structure within the subarachnoid space could not be observed. Morphological studies of subarachnoid trabeculae in the OAC will be improved in the future, and their potential role in CSF flow will be investigated.

## Conclusions

At the OCJ, there is an enlarged subarachnoid space dorsal to both the medulla oblongata and the upper portion of the cervical spinal cord, extending from the foramen magnum to the upper border of the axis. This newly described broad subarachnoid space, which is named the “occipito-atlantal cistern”, becomes a hub for the diffusion of CSF from the cisterna magna to the subarachnoid space of the upper cervical spine. And the present study adds a knowledge base to the structural and functional studies of the occipito-cervical junction.

## Data Availability

All data generated or analyzed during this study are included in this article. All the data was obtained and documented via the described experiments.
